# Modified generalized Weibull distribution: theory and applications

**DOI:** 10.1038/s41598-023-38942-9

**Published:** 2023-08-07

**Authors:** Mustafa S. Shama, Amirah Saeed Alharthi, Fatimah A. Almulhim, Ahmed M. Gemeay, Mohammed Amine Meraou, Manahil SidAhmed Mustafa, Eslam Hussam, Hassan M. Aljohani

**Affiliations:** 1 Department of Basic Sciences, CFY, King Saud University, Riyadh, 12373 Saudi Arabia; 2 Department of Mathematics and Statistics, Osim Higher Institute of Administrative Science, Osim, 12961 Egypt; 3 Department of Mathematics and Statistics, College of Science, Taif University, P.O. Box 11099, Taif, 21944 Saudi Arabia; 4 Department of Mathematical Sciences, College of Sciences, Princess Nourah bint Abdulrahman University, P.O. Box 84428, Riyadh, 11671 Saudi Arabia; 5 Department of Mathematics, Faculty of Science, Tanta University, Tanta, 31527 Egypt; 6 Laboratory of Statistics and Stochastic Processes, University of Djillali Liabes, BP 89, 22000 Sidi Bel Abbès, Algeria; 7 Department of Statistics, Faculty of Science, University of Tabuk, Tabuk, Saudi Arabia; 8 Department of Mathematics, Faculty of Science, Helwan University, Cairo, Egypt

**Keywords:** Mathematics and computing, Statistics

## Abstract

This article presents and investigates a modified version of the Weibull distribution that incorporates four parameters and can effectively represent a hazard rate function with a shape resembling a bathtub. Its significance in the fields of lifetime and reliability stems from its ability to model both increasing and decreasing failure rates. The proposed distribution encompasses several well-known models such as the Weibull, extreme value, exponentiated Weibull, generalized Rayleigh, and modified Weibull distributions. The paper derives key mathematical statistics of the proposed distribution, including the quantile function, moments, moment-generating function, and order statistics density. Various mathematical properties of the proposed model are established, and the unknown parameters of the distribution are estimated using different estimation techniques. Furthermore, the effectiveness of these estimators is assessed through numerical simulation studies. Finally, the paper applies the new model and compares it with various existing distributions by analyzing two real-life time data sets.

## Introduction

Statistical models are crucial in comprehending and predicting real-world phenomena. In numerous applications, it becomes necessary to utilize enhanced versions of well-established distributions. These new distributions offer greater flexibility when it comes to simulating real-world data with high skewness and kurtosis. Among the advantages of the new distribution is its suitability for various fields, including medical, financial, and engineering applications. Selecting the most appropriate statistical model for data analysis is both critical and challenging. For further exploration on the topic of distributions, I recommend referring to the following references: Almongy et al.^1^, Shafiq et al.^2^, and Meriem et al.^3^. These sources provide additional insights and information.

The Weibull distribution is extensively employed in the analysis of lifetime data and has demonstrated notable efficacy in capturing failure rates that display monotonic patterns. Its density shapes, which manifest as either right or left-skewed, render it well-suited for survival and reliability analysis. Nevertheless, the Weibull model is inadequate for accurately representing non-monotonic failure rates, such as those characterized by hazard functions exhibiting bathtub-shaped or upside-down bathtub-shaped patterns. To address this limitation, researchers have developed enhanced versions of the Weibull distribution that can accurately accommodate different hazard function shapes to represent complex failure models accurately. Xie and Lai^4^ introduced the additive Weibull distribution, incorporating a bathtub-shaped hazard function. Bebbington et al.^5^ proposed the flexible Weibull distribution, which modifies the hazard function to exhibit an increasing pattern followed by a bathtub shape. Lai et al.^6^ presented a new Weibull distribution model with three parameters and a bathtub-shaped hazard function.

Notwithstanding the progress made in the field, numerous prevailing models exhibit limited flexibility and may not yield optimal fits when applied to real-world data in engineering and related domains. To address this issue, researchers have employed diverse techniques to develop alternative distributions that enhance the flexibility of existing models. One approach involves generating a new distribution by combining two cumulative hazard rate (CHR) functions through a mixture model. It can be written as below:

1 $$\begin{aligned} H\left( x\right) =H_{1} \left( x\right) +H_{2} \left( x\right) , \end{aligned}$$

with $H\left( x\right)$ denoted the cumulative hazard rate function satisfies the following conditions

1. $$\mathop {\lim }\limits _{x\rightarrow 0} H\left( x\right) =0$$,
2. $$\mathop {\lim }\limits _{x\rightarrow \infty } H\left( x\right) =\infty$$,
3. $H\left( x\right)$ is a differentiable non-negative and non-decreasing.

By using Eq. (1), the generated cumulative density function (cdf) and probability density function (pdf) are, respectively, given by

2 $$\begin{aligned} G\left( x\right) =1-e^{-H_{1} \left( x\right) -H_{2} \left( x\right) }, \end{aligned}$$

3 $$\begin{aligned} g\left( x\right) =\left( h_{1} \left( x\right) +h_{2} \left( x\right) \right) e^{-H_{1} \left( x\right) -H_{2} \left( x\right) }. \end{aligned}$$

Some generalized distributions generated according to (2) and (3) are listed in Table 1.

**Table 1:** Some generalized distributions of a mixture of the two chr functions.

S.N	Name of the distribution		
	$H_{1} \left( x\right)$	$H_{2} \left( x\right)$	$H\left( x\right)$
1	Weibull	Weibull	Additive Weibull^4^
2	Weibull	Modified Weibull^6^	New modified Weibull^7^
3	Exponential	Weibull	Modified Weibull^8^
4	Exponential	Exponential	Modified exponential^9^

Bagdonavicius and Nikulin^10^ proposed an extension of the Weibull distribution, namely power generalized Weibull (PGW) distribution, and its cdf and pdf can be described as

4 $$\begin{aligned} F\left( x\right) =1-\exp \left( 1-\left( 1+\lambda x^{\theta } \right) ^{\alpha } \right) ,~x, \alpha ,\lambda ,\theta \mathrm{>}0, \end{aligned}$$

and

5 $$\begin{aligned} f\left( x\right) =\alpha \lambda \theta \left( 1+\lambda x^{\theta } \right) ^{\alpha -1} \exp \left( 1-\left( 1+\lambda x^{\theta } \right) ^{\alpha } \right) , \end{aligned}$$

and the relationship between cdf and pdf is given by

6 $$\begin{aligned} f\left( x\right) =\alpha \lambda \theta \left( 1+\lambda x^{\theta } \right) ^{\alpha -1} (1-F\left( x\right) ), \end{aligned}$$

respectively, where $\alpha$ and $\theta$ are two shape parameters and $\lambda$ is a scale parameter. PGW distribution contains constant, monotone (increasing or decreasing), bathtub-shaped, and unimodal hazard shapes. For more details about this extension, see, for example, Bagdonavicius and Nikulin^11^, Voinov et al.^12^, and Kumar and Dey^13^.

In this research article, we introduce a novel statistical model called the modified power generalized Weibull (MPGW) distribution. Four parameters characterize the MPGW distribution and exhibit several significant properties. This distribution’s probability density function (pdf) can assume different forms, including constant, monotonic (increasing or decreasing), and unimodal. Moreover, the hazard rate function (hrf) associated with the MPGW distribution can take on various shapes, such as constant, monotonic, bathtub, and upside-down bathtub.

We investigate several mathematical properties of the MPGW distribution and explore its applicability in different contexts. To estimate the model parameters, we employ various estimation techniques, including maximum likelihood estimation (MLE), the maximum product of spacing (MPS), least square estimators (LSE), and Cramer-von Mises estimators (CVE). These estimation methods enable us to determine the most suitable parameter values for the MPGW distribution based on the available data.

The proposed distribution was used in many fields of science such as engineering and bio-sciences as it can model many kinds of data because of the distribution’s great flexibility. For more details about similar papers see^12,14^ The rest of this paper is structured as follows. Section “The formulation of the MPGW distribution” described the new MPGW model and provided different distributional properties. Further, numerous statistical properties for the proposed distribution were introduced in Section “Statistical properties”. In Section “Estimation methods”, we established different estimation procedures for the unknown parameters of the suggested distribution. Monte Carlo simulation studies are performed in Section “Numerical simulation” to compare the proposed estimators. Finally, in Section “Real data analysis”, two real data sets defined by the survival field are analyzed for validation purposes, and we conclude the article in Section “Conclusion”.

### Main contribution and novelty

This research paper presents a noteworthy advancement in the field of probability distributions by introducing a novel four-parameter generalization of the Weibull distribution. The proposed generalization offers the ability to model a hazard rate function that exhibits a bathtub-shaped pattern. The bathtub-shaped hazard rate function is of great interest in various domains, as it accurately captures the characteristics of failure rates observed in certain real-world scenarios. To evaluate the efficacy of the newly proposed model, we conducted an empirical investigation using two distinct real-life time data sets. These data sets were carefully selected to encompass diverse applications and ensure the generalizability of the findings. We could assess the model’s effectiveness in practical applications by employing the proposed four-parameter generalized Weibull distribution and comparing its performance with several existing distributions. Through a comprehensive analysis of the results, valuable insights were obtained regarding the capabilities and advantages of the novel four-parameter generalized Weibull distribution when applied to real-world data sets. The comparison of the proposed model with existing distributions provided a rigorous evaluation framework, enabling a thorough understanding of its performance in different scenarios. This study contributes to the existing body of knowledge by demonstrating the applicability and usefulness of the new distribution in capturing the complexities of time-to-failure data.

## The formulation of the MPGW distribution

The MPGW distribution is generated by using $H_{1} \left( x\right)$ of the PGW distribution and $H_{2} \left( x\right)$ of the exponential distribution in Eqs. (2) and (3). Its cdf and pdf can be defined as the following

7 $$\begin{aligned} G\left( x\right) =1-\textrm{e}^{1-\left( 1+\lambda x^{\theta } \right) ^{\alpha } -\beta x},\, x\mathrm{>}0, \end{aligned}$$

8 $$\begin{aligned} g\left( x\right) =\left( \beta +\alpha \theta \lambda x^{\theta -1} \left( 1+\lambda x^{\theta } \right) ^{\alpha -1} \right) \textrm{e}^{1-\left( 1+\lambda x^{\theta } \right) ^{\alpha } -\beta x}, \end{aligned}$$

and the relationship between cdf and pdf can be written as

9 $$\begin{aligned} g\left( x\right) =\left( \beta +\alpha \theta \lambda x^{\theta -1} \left( 1+\lambda x^{\theta } \right) ^{\alpha -1} \right) (1-G\left( x\right) ), \end{aligned}$$

where $\theta >0$, $\lambda ,\alpha ,\beta \ge 0$ such that $\lambda +\beta >0$ and $\alpha +\beta >0$.

The hazard rate function (hrf) of the MPGW model can be expressed as

10 $$\begin{aligned} h\left( x\right) =\beta +\alpha \theta \lambda x^{\theta -1} \left( 1+\lambda x^{\theta } \right) ^{\alpha -1}. \end{aligned}$$

Table 2 summarized several well-known lifetime distributions from the newly suggested distribution, which is quite flexible.

**Table 2:** Some special models of the MPGW distribution.

Parameters				Distribution
$\lambda$	$\alpha$	$\beta$	$\theta$	
–	–	0	–	Power generalized Weibull (PGW)^11^
–	1	–	–	Modified Weibull (MW)^8^
–	–	–	1	Modified Nadarajah–Haghighi (MNH) (new)
–	–	0	1	Nadarajah–Haghighi (NH)^15^
–	1	–	2	linear failure rate (LFR)^16^
–	1	0	–	Weibull (W)
–	1	0	2	Rayleigh (R)
0	–	–	–	Exponential (E)
–	0	–	–	
–	1	0	1	

## Statistical properties

In this part of the study, we provided some mathematical properties of the MPGW distribution, especially moments, skewness, kurtosis, and asymmetry.

### Behavior of the pdf of the MPGW distribution

The pdf limits of the MPGW distribution are

$$\begin{aligned} \mathop {\lim }\limits _{x\rightarrow 0^{+} } f\left( x\right) =\left\{ \begin{array}{l} {\infty \, \, \, \, \, \, \, \, \, \, \, \, \, \, \, \, \, \, \, \, \, \theta \mathrm{<}1} \\ {\beta +\alpha \lambda \, \, \, \, \, \, \, \, \, \, \theta \mathrm{=}1,\, \, \, \, \, \, f\left( \infty \right) =0\, } \\ {\beta \, \, \, \, \, \, \, \, \, \, \, \, \, \, \, \, \, \, \, \, \, \theta \mathrm{>}1} \end{array}\right. . \end{aligned}$$

**Figure 1:**
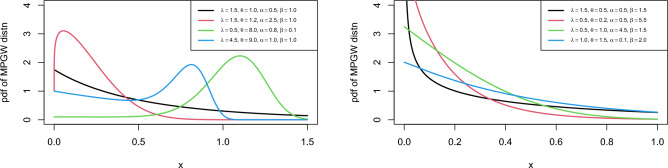
Plot for PDF of the MPGW model for different values of the parameters.

From the pdf of the MPGW distribution, the first derivative of the pdf is

$$\begin{aligned}{} & {} f\mathrm{{'} }\left( x\right) =-\frac{\psi \left( x\right) }{h\left( x\right) } f\left( x\right) ,\\{} & {} \psi \left( x\right) =\left( \beta +\alpha \theta \lambda x^{\theta -1} \left( 1+\lambda x^{\theta } \right) ^{\alpha -1} \right) ^{2} +\alpha \theta \lambda x^{\theta -2} \left( 1+\lambda x^{\theta } \right) ^{\alpha -2} \left( 1-\theta -\lambda \left( \alpha \theta -1\right) x^{\theta } \right) , \end{aligned}$$

where $\psi \left( x\right) =\left( h\left( x\right) \right) ^{2} -h\mathrm{{'} }\left( x\right)$. It is clear that $f\mathrm {{'} }\left( x\right)$ and $\psi \left( x\right)$ have the same sign, and $\psi \left( x\right)$ has not an explicit solution. Therefore, we can discuss the following special cases which depend on $\theta$ and $\alpha$:

- *Case 1*: For $\theta \le 1$ and $\alpha \theta \le 1$, $\psi \left( x\right)$ is negative which means $f\left( x\right)$is decreasing in *x*
- *Case 2*: For $\theta =1$, $\psi \left( x\right)$ reduces to
  $$\begin{aligned} \left( \alpha -1\right) \alpha \lambda ^{2} \left( 1+x\lambda \right) ^{\alpha -2} -\left( \beta +\alpha \lambda \left( 1+x\lambda \right) ^{\alpha -1} \right) ^{2}, \end{aligned}$$
  which has no solution for $\alpha \le 1$ and the pdf becomes decreasing for all *x*.
- *Case 3*: For $\alpha =1$, $\psi \left( x\right)$ reduces to
  $$\begin{aligned} \theta \lambda \left( \theta -1\right) x^{\theta -2} -\left( \beta +\theta \lambda x^{\theta -1} \right) ^{2}, \end{aligned}$$
  which has no solution for $\theta \le 1$ and the pdf becomes decreasing for all *x*.
- *Case 4*: For$\beta =0$ and $\theta =1$, $\psi \left( x\right)$ reduces to
  $$\begin{aligned} \alpha \lambda ^{2} \left( 1+\lambda x\right) ^{-2+\alpha } \left( \alpha \left( 1-\left( 1+\lambda x\right) ^{\alpha } \right) -1\right) , \end{aligned}$$
  which has a solution for $\alpha \mathrm {>}1$, therefore the mode (M) becomes
  $$\begin{aligned} M=\frac{\left( 1-1/\alpha \right) ^{1/\alpha } -1}{\lambda }. \end{aligned}$$
  *Case 5*: For $\alpha =1$and $\beta =0$, $\psi \left( x\right)$ reduces to
  $$\begin{aligned} \theta \lambda x^{\theta -2} \left( \theta \left( 1-x^{\theta } \lambda \right) -1\right) , \end{aligned}$$
  which has a solution for $\theta \mathrm {>}1$, therefore the mode becomes
  $$\begin{aligned} M=\left( \left( \theta -1\right) /\theta \lambda \right) ^{1/\theta }. \end{aligned}$$
  *Case 6*: For$\alpha =1$, $\beta =0$ and $\theta =2$, $\psi \left( x\right)$ reduces to
  $$\begin{aligned} 2\lambda \left( 1-2x^{2} \lambda \right) , \end{aligned}$$
  in this case, the mode becomes
  $$\begin{aligned} M=1/\sqrt{2\lambda }. \end{aligned}$$
  For different parameter values, Fig. 1 depicts the pdf plots of MPGW distribution. The graphs show that the pdf of MPGW is decreasing and uni-modal which gives our proposed model the superiority for analyzing lifetime data.

### Behavior of the hazard rate function of the MPGW distribution

The hrf limits of the MPGW distribution are

$$\mathop {\lim }\limits _{x\rightarrow 0^{+} } h\left( x\right) =\left\{ \begin{array}{l} {\infty \, \, \, \, \, \, \, \, \, \, \, \, \, \, \, \, \, \, \, \, \, \theta \mathrm{<}1} \\ {\beta +\alpha \lambda \, \, \, \, \, \, \, \, \, \, \theta \mathrm{=}1,} \\ {\beta \, \, \, \, \, \, \, \, \, \, \, \, \, \, \, \, \, \, \, \, \, \theta \mathrm{>}1} \end{array}\right.$$

$$\mathop {\lim }\limits _{x\rightarrow \infty } h\left( x\right) =\left\{ \begin{array}{l} {\beta \, \, \, \, \, \, \, \, \, \, \, \, \, \, \, \, \, \alpha \theta \mathrm{<}1} \\ {\infty \, \, \, \, \, \, \, \, \, \, \, \, \, \, \, \, \, \alpha \theta \mathrm{>}1} \end{array}\right. ,$$

and

$$\mathop {\lim }\limits _{x\rightarrow \infty } h\left( x\right) =\left\{ \begin{array}{l} {\beta \, \, \, \, \, \, \, \, \, \, \, \, \, \, \, \, \, \, \, \, \, \, \, \alpha \theta \mathrm{=}1,\, \, \theta \mathrm{<1}} \\ {\beta + \lambda \, \, \, \, \, \, \, \, \, \, \, \, \alpha \theta \mathrm{=}1,\, \, \theta \mathrm{=1}} \\ {\infty \, \, \, \, \, \, \, \, \, \, \, \, \, \, \, \, \, \, \, \, \, \, \, \alpha \theta \mathrm{=}1,\, \, \theta \mathrm{>1}} \end{array}\right. .$$

The study of the shape of the hrf needs an analysis of the first derivative $h\mathrm {{'} }\left( x\right)$ and it can be described as

11 $$\begin{aligned} h\mathrm {{'} }\left( x\right) =\alpha \theta \lambda x^{\theta -2} \left( 1+x^{\theta } \lambda \right) ^{\alpha -2} \eta \left( x\right) , \end{aligned}$$

where $\eta \left( x\right) =\theta -1+\lambda \left( \alpha \theta -1\right) x^{\theta }$. Clearly, $h\mathrm {{'} }\left( x\right)$ and $\eta \left( x\right)$ have the same sign and $\eta \left( x\right)$ has critical value at the point

$$\begin{aligned}x^{*} =\left( \frac{1-\theta }{\left( \alpha \theta -1\right) \lambda } \right) ^{1/\theta } \end{aligned}$$

**Figure 2:**
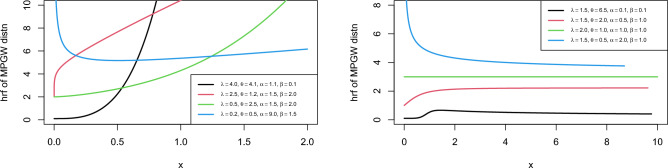
Plot for PDF of the MPGW distribution for different values of the parameters.

From $\eta \left( x\right)$, it can be noted that the hrf has different shapes written as:

- *Case1: *$$\alpha \theta \mathrm {>}1$$.
  1. If $\theta \ge 1$, then $h\mathrm {{'} }\left( x\right) >0$ and $h\left( x\right)$ are monotonically increasing.
  2. If $\theta \mathrm {<}1$, then the hrf is decreasing for $x\mathrm {<}x^{*}$ and increasing for$x\mathrm {>}x^{*}$. Hence, the hrf has a bathtub shape.
- *Case2: *$$\alpha \theta \mathrm {<}1$$.
  1. If $\theta \le 1$, then $h\mathrm {{'} }\left( x\right) \mathrm {<}0$ and $h\left( x\right)$ are monotonically decreasing.
  2. If $\theta \mathrm {>}1$, this means $\mathrm {0<}\alpha \mathrm {<}1$and $\mathrm {1<}\theta \mathrm {<}1/\alpha$, then the hrf is increasing for $x\mathrm {<}x^{*}$and the hrf is decreasing for $x\mathrm {>}x^{*}$. Hence, the hrf has an upside-down bathtub shape.
- *Case3: *$$\alpha \theta =1$$.
  1. $h\mathrm {{'} }\left( x\right) \mathrm {=}0$ and $h\left( x\right)$ are constant when $\theta$.
  2. $h\mathrm {{'} }\left( x\right) >0$ and $h\left( x\right)$ are monotonically increasing where $\theta \mathrm {>}1$.
  3. $h\mathrm {{'} }\left( x\right) \mathrm {<}0$ and $h\left( x\right)$ are monotonically decreasing where $\theta \mathrm {<}1$.
  Figure 2 displays the plot of hrf of MPGW model for multiple parameter values. The plots of hrf of MPGW are more efficient in modeling lifetime data.

### Moments

#### Theorem 1

For any $r\in N$, the rth raw moment of the MPGW model can be written as

$$\begin{aligned} \mu _{r}^{\mathrm{{'} }} =\left\{ \begin{array}{l} {\sum _{i=0}^{\infty }\frac{\left( -1\right) ^{i} \beta ^{i} e}{i\mathrm{!}} \left[ \beta I\left( r,i\right) +\alpha \theta \lambda K\left( r,i\right) \right] \, \, \, \, \, \, for\, \lambda ,\alpha ,\beta \mathrm{>}0} \\ {\, \, \alpha \theta \lambda eK\left( r,0\right) \, \, \, \, \, \, \, \, \, \, \, \, \, \, \, \, \, \, \, \, \, \, \, \, \, \, \, \, \, \, \, \, \, \, \, \, \, \, \, \, \, \, \, \, \, \, \, \, \, \, \, \, \, \, for \beta \mathrm{=}0,\lambda ,\alpha \mathrm{>}0} \\ {\frac{\Gamma \left( r+1\right) }{\beta ^{r} } \, \, \, \, \, \, \, \, \, \, \, \, \, \, \, \, \, \, \, \, \, \, \, \, \, \, \, \, \, \, \, \, \, \, \, \, \, \, \, \, \, \, \, \, \, \, \, \, \, \, \, \, \, \, \, \, \, \, \, \, \, \, \, \, for\lambda \, or\, \alpha \mathrm{=}0,\, \beta \mathrm{>}0\, \, } \end{array}\right. \, \, \,. \end{aligned}$$

#### Proof

By the pdf (8) and the definition of the rth raw moment, we have

12 $$\begin{aligned} \mu _{r}^{\mathrm{{'} }} =\int _{0}^{\infty }x^{r} \left( \beta +\alpha \theta \lambda x^{\theta -1} \left( 1+\lambda x^{\theta } \right) ^{\alpha -1} \right) \textrm{e}^{1-\left( 1+\lambda x^{\theta } \right) ^{\alpha } -\beta x} dx. \end{aligned}$$

In the general case, we suppose that $\lambda$, $\alpha$ and $\beta \mathrm{>}0$. Using the following expansion of $\textrm{e}^{-\beta x}$given by

$$\begin{aligned} \textrm{e}^{-\beta x} =\sum _{i=0}^{\infty }\frac{\left( -1\right) ^{i} \beta ^{i} x^{i} }{i\mathrm{!}}, \end{aligned}$$

then Eq. (12) is rewritten as

13 $$\begin{aligned} \mu _{r}^{\mathrm{{'} }} =\sum _{i=0}^{\infty }\frac{\left( -1\right) ^{i} \beta ^{i} e}{i\mathrm{!}} \int _{0}^{\infty }x^{r+i} \left( \beta +\alpha \theta \lambda x^{\theta -1} \left( 1+\lambda x^{\theta } \right) ^{\alpha -1} \right) \textrm{e}^{-\left( 1+\lambda x^{\theta } \right) ^{\alpha } } dx. \end{aligned}$$

Let $I\left( r,i\right) =\int _{0}^{\infty }x^{r+i} \textrm{e}^{-\left( 1+\lambda x^{\theta } \right) ^{\alpha } } dx$ and $u=\left( 1+\lambda x^{\theta } \right) ^{\alpha }$, we have

$$\begin{aligned} I\left( r,i\right) =\frac{1}{\alpha \theta \lambda ^{\left( r+i+1\right) /\theta } } \int _{1}^{\infty }u^{\left( r+i+1\right) /\alpha \theta -1} \left( 1-u^{-1/\alpha } \right) ^{\left( r+i+1\right) /\theta -1} \textrm{e}^{-u} du. \end{aligned}$$

By using the expansion of $\left( 1-u^{-1/\alpha } \right) ^{\left( r+i+1\right) /\theta -1}$ where $\left| u^{-1/\alpha } \right| \mathrm{<}1$, above integral is described as

$$\begin{aligned} I\left( r,i\right) =\frac{1}{\alpha \theta \lambda ^{\left( r+i+1\right) /\theta } } \int _{1}^{\infty }u^{\left( i+1\right) /\alpha -1} \sum _{j=0}^{\infty }\left( \begin{array}{l} {\left( r+i+1\right) /\theta -1} \\ {\, \, \, \, \, \, \, \, \, \, \, \, \, \, \, \, j} \end{array}\right) \left( -1\right) ^{\left( r+i+1\right) /\theta -j-1} \textrm{e}^{-u} du. \end{aligned}$$

Hence, after some algebra, we get

14 $$\begin{aligned} I\left( r,i\right) =\frac{1}{\alpha \theta \lambda ^{\left( r+i+1\right) /\theta } } \sum _{j=0}^{\infty }\left( \begin{array}{l} {\left( r+i+1\right) /\theta -1} \\ {\, \, \, \, \, \, \, \, \, \, \, \, \, \, \, \, j} \end{array}\right) \left( -1\right) ^{\left( r+i+1\right) /\theta -j-1} \Gamma \left( \left( i+1\right) /\alpha ,1\right) , \end{aligned}$$

let $k\left( r,i\right) =\int _{0}^{\infty }x^{r+i+\theta -1} \left( 1+\lambda x^{\theta } \right) ^{\alpha -1} \textrm{e}^{-\left( 1+\lambda x^{\theta } \right) ^{\alpha } } dx$ and $u=\left( 1+\lambda x^{\theta } \right) ^{\alpha }$, we have

$$\begin{aligned} K\left( r,i\right) =\frac{1}{\alpha \theta \lambda ^{\left( r+i\right) /\theta -1} } \int _{1}^{\infty }u^{\left( r+i\right) /\alpha \theta } \left( 1-u^{-1/\alpha } \right) ^{\left( r+i\right) /\theta } \textrm{e}^{-u} du. \end{aligned}$$

Hence, after some algebra, we obtain

15 $$\begin{aligned} K\left( r,i\right) =\frac{1}{\alpha \theta \lambda ^{\left( r+i\right) /\theta +1} } \sum _{l=0}^{\infty }\left( \begin{array}{l} {\left( r+i\right) /\theta } \\ {\, \, \, \, \, \, \, \, \, l} \end{array}\right) \left( -1\right) ^{\left( r+i\right) /\theta -l} \Gamma \left( l/\alpha +1,1\right) , \end{aligned}$$

finally, substituting (14) and (15) into (13), we have

$$\begin{aligned} \mu _{r}^{\mathrm{{'} }} =\sum _{i=0}^{\infty }\frac{\left( -1\right) ^{i} \beta ^{i} e}{i\mathrm{!}} \left[ \beta I\left( r,i\right) +\alpha \theta \lambda K\left( r,i\right) \right] , \end{aligned}$$

which completes the proof. $\square$

According to the results given in theorem 3, the mean and the variance of the proposed model, respectively, are $\mu =\mu _{1}^{\mathrm{{'} }}$ and $\sigma ^{2} =\mu _{2}^{\mathrm{{'} }} -\mu ^{2}$. As well as the measures of skewness, kurtosis, and  asymmetry of the MPGW are given, respectively, by

$$\begin{aligned} \beta _{1} =\frac{\left( \mu _{3}^{\mathrm{{'} }} -3\mu _{2}^{\mathrm{{'} }} \mu +2\mu ^{3} \right) ^{2} }{\left( \mu _{2}^{\mathrm{{'} }} -\mu ^{2} \right) ^{3} }, \\\beta _{2} =\frac{\mu _{4}^{\mathrm{{'} }} -4\mu _{3}^{\mathrm{{'} }} \mu +6\mu _{2}^{\mathrm{{'} }} \mu ^{2} -3\mu ^{4} }{\left( \mu _{2}^{\mathrm{{'} }} -\mu ^{2} \right) ^{2} }, \end{aligned}$$

and

$$\begin{aligned} \beta _{3} =\frac{\mu _{3}^{\mathrm{{'} }} -3\mu _{2}^{\mathrm{{'} }} \mu +2\mu ^{3} }{\left( \mu _{2}^{\mathrm{{'} }} -\mu ^{2} \right) ^{3/2} }. \end{aligned}$$

Table 3 shows some necessary MPGW measures for various parameter combinations computed using the R program.

**Table 3:** Some statistical measures for MPGW using varied parameter values.

Initial values				Mean	Variance	$\beta _1$	$\beta _1$	${\beta _3}$
$\alpha =0.5$	$\lambda =0.5$	$\theta =0.4$	$\beta =0.6$	1.317838	2.292988	4.886313	10.27683	2.210501
			$\beta =1.4$	0.6005073	0.4433549	4.615957	9.889532	2.148478
		$\theta =1.2$	$\beta =0.6$	1.212822	1.466835	4.373271	9.787101	2.091237
			$\beta =1.4$	0.617936	0.3741446	4.017766	9.10745	2.004437
	$\lambda =1.5$	$\theta =0.4$	$\beta =0.6$	0.9286623	1.67044	6.959431	13.26558	2.638073
			$\beta =1.4$	0.4568996	0.3493235	6.031006	11.92106	2.455811
		$\theta =1.2$	$\beta =0.6$	0.868915	0.8067142	5.272788	11.4164	2.296255
			$\beta =1.4$	0.5099617	0.2601584	4.419819	9.872812	2.102337
*$\alpha =1.0$	$\lambda =0.5$	$\theta =0.4$	$\beta =0.6$	0.9929894	1.672137	6.319516	12.41974	2.513865
			$\beta =1.4$	0.4912752	0.3591074	5.520644	11.22754	2.349605
		$\theta =1.2$	$\beta =0.6$	0.884102	0.655516	2.983307	7.295088	1.727225
			$\beta =1.4$	0.5313037	0.2557791	3.37493	7.938339	1.837098
	$\lambda =1.5$	$\theta =0.4$	$\beta =0.6$	0.4069533	0.5349628	14.19162	24.82229	3.767177
			$\beta =1.4$	0.2506932	0.1608244	10.33383	18.61577	3.214627
		$\theta =1.2$	$\beta =0.6$	0.496919	0.1910405	2.647061	6.756577	1.626979
			$\beta =1.4$	0.3656575	0.1113983	2.953406	7.246701	1.718548
$\alpha =1.5$	$\lambda =0.5$	$\theta =0.4$	$\beta =0.6$	0.7150675	1.068093	8.18407	15.3267	2.860781
			$\beta =1.4$	0.3912617	0.2697455	6.666465	12.97332	2.58195
		$\theta =1.2$	$\beta =0.6$	0.6835784	0.3333469	1.94893	5.440328	1.396041
			$\beta =1.4$	0.4602635	0.1742717	2.615656	6.533268	1.617299
	$\lambda =1.5$	$\theta =0.4$	$\beta =0.6$	0.160819	0.1027797	21.3466	37.79339	4.620238
			$\beta =1.4$	0.1242004	0.05074849	15.55551	27.49139	3.944048
		$\theta =1.2$	$\beta =0.6$	0.3446051	0.07488582	1.537765	4.803849	1.240066
			$\beta =1.4$	0.2801202	0.05536821	1.907145	5.374306	1.380994

From the values of Table 3 it can be deduced that

1. If $\alpha$ increases and for fixed $\beta$, $\lambda$ and $\theta$, the values of Mean and Variance of the suggested MPGW model tend to decrease, while the values of $\beta _1$, $\beta _2$ and $\beta _3$ are increasing. The same result for $\lambda$ with fixed $\alpha$, $\beta$ and $\theta$.
2. For fixed values of $\alpha$, $\lambda$ and $\theta$ and for $\beta$ augment, all values of Mean, Variance, $\beta _1$, $\beta _2$ and $\beta _3$ of the MPGW model are decrease..
3. The MPGW distribution is a flexible model for explaining more data sets.

## Estimation methods

Here, we considered four estimation techniques for constructing the estimation of the unknown parameters for MPGW model. The determination of the estimate parameters using different procedures has been made available to various authors such as^17–19^.

### Maximum likelihood estimation and its asymptotics

Let $\{x_1, \ldots , x_n\}$ be a a random sample coming from MPGW$(\alpha , \beta , \lambda , \theta )$. Then, the corresponding log-likelihood function is described by

16 $$\begin{aligned} {\mathcal {L}}{\mathcal {L}}(\Theta )= & {} \displaystyle \sum _{i=1}^{n} \ln f(x_i)=n\ln \beta +n\ln (\alpha \theta \beta )+(\theta -1)\displaystyle \sum _{i=1}^{n}\ln x_i+(\alpha -1)\displaystyle \sum _{i=1}^{n}\ln (1+\lambda x_i^\theta ) \nonumber \\{} & {} \quad + n-\displaystyle \sum _{i=1}^{n}(1+\lambda x_i^\theta )^\alpha -\beta \displaystyle \sum _{i=1}^{n} x_i. \end{aligned}$$

with $\Theta =(\alpha , \beta , \lambda , \theta )$. Consequently, with respect to $\alpha , \beta , \lambda$, and $\theta$ and by taking the derivatives of (16), we can be determined the estimates ${\hat{\alpha }}_{MLE}$, ${\hat{\beta }}_{MLE}$, ${\hat{\lambda }}_{MLE}$ and ${\hat{\theta }}_{MLE}$ and these estimates are given respectively by

17 $$\begin{aligned}{} & {} \dfrac{\partial {\mathcal {L}}{\mathcal {L}}}{\partial \alpha }=\dfrac{n}{\alpha }+\displaystyle \sum _{i=1}^{n}\ln (1+\lambda x_i^\theta )-\ln \bigg (\displaystyle \sum _{i=1}^{n}(1+\lambda x_i^\theta )\bigg ) \exp \bigg [\alpha \ln \bigg (\displaystyle \sum _{i=1}^{n}(1+\lambda x_i^\theta )\bigg )\bigg ], \end{aligned}$$

18 $$\begin{aligned}{} & {} \dfrac{\partial {\mathcal {L}}{\mathcal {L}}}{\partial \beta }= \dfrac{2n}{\beta }-\displaystyle \sum _{i=1}^{n}x_i, \end{aligned}$$

19 $$\begin{aligned}{} & {} \dfrac{\partial {\mathcal {L}}{\mathcal {L}}}{\partial \lambda }= (\alpha -1)\displaystyle \sum _{i=1}^{n}\dfrac{x_i^{\theta }}{1+\lambda x_i^{\theta }}-\alpha \displaystyle \sum _{i=1}^{n}x_i^{\theta }(1+\lambda x_i^{\theta })^{\alpha -1}, \end{aligned}$$

and

20 $$\begin{aligned} \dfrac{\partial {\mathcal {L}}{\mathcal {L}}}{\partial \theta }=\dfrac{n}{\theta }+\displaystyle \sum _{i=1}^{n}\ln x_i +\lambda (\alpha -1) \displaystyle \sum _{i=1}^{n}\dfrac{\ln x_i e^{\theta \ln x_i}}{1+\lambda x_i^{\theta }} -\alpha \lambda \displaystyle \sum _{i=1}^{n} \ln x_i e^{\theta \ln x_i} (1+\lambda x_i^{\theta })^{\alpha -1}. \end{aligned}$$

These estimates can be solved numerically using various approach methods, including Newton Raphson, bisection, or fixed point methods.

### Least square estimation

Let $x_1,\ldots ,x_n$ be a random sample from MPGW$(\alpha , \beta , \lambda , \theta )$ and $x_{1:n}<\cdots <x_{n:n}$ represent the order statistics of the random sample from the MPGW model. The least-square estimator (LSE) which introduced by^20^) of $\alpha , \beta , \lambda , \theta$, noted by ${\hat{\alpha }}_{LSE}$, ${\hat{\beta }}_{LSE}$, ${\hat{\lambda }}_{LSE}$ and ${\hat{\theta }}_{LSE}$) can be described by minimizing

$$\begin{aligned} \displaystyle \sum _{i=1}^{n}\bigg [ F(x_{i:n}|\Theta )-\dfrac{i}{n+1}\bigg ]^{2}. \end{aligned}$$

### Maximum product of spacings

For $x_1\le \cdots \le x_n$ representing the ordered statistics random sample from MPGW distribution, the maximum product of the spacings estimation (MPS) estimators of the proposed model resulted by maximizing the following equation

21 $$\begin{aligned} {\mathcal {M}}{\mathcal {P}}(\Theta )= \left[ \displaystyle \prod _{i=1}^{n+1}L_{i}(\Theta )\right] ^{1/(n+1)}, L_{i}(\Theta )=F(x_{i:n}|\Theta )-F(x_{i-1:n}|\Theta ). \end{aligned}$$

### Cramer-von Mises minimum distance estimators

The Cramer-von Mises-type minimum distance estimators (CVEs) ${\hat{\alpha }}_{CVE}$, ${\hat{\beta }}_{CVE}$, ${\hat{\lambda }}_{CVE}$ and ${\hat{\theta }}_{CVE}$ of $\alpha , \beta , \lambda , \theta$ are described respectively by minimizing

22 $$\begin{aligned} {\mathcal {C}}{\mathcal {R}}(\Theta )=\dfrac{1}{12n}+\sum _{i=1}^{n}\bigg [ F(x_{i:n}|\Theta )-\dfrac{2i-1}{2n}\bigg ]^{2}. \end{aligned}$$

## Numerical simulation

Here in this part of the work, we performed some results from simulation experiments so that you may assess how well the various estimating techniques provided in Section “Estimation methods” using different sample sizes, $n = \{100, 300, 500, 700, 1000\}$ and different sets of initial parameters. After repeating the process $K = 1000$, we generate different random samples from the suggested model. The following algorithm can be easily used to generate samples from the MPGW distribution

1. Step 1: Generate *u* from U(0,1).
2. Step 2: Generate *x* as *x* is the solution of equation $1-\textrm{e}^{1-\left( 1+\lambda x^{\theta } \right) ^{\alpha } -\beta x}=u$.

Further, we compute the average values of biases (AB), mean square errors (MSEs), and mean relative errors (MREs) by the following equations

$$\begin{aligned} |BIAS|= \frac{1}{K}\sum _{i=1}^{K}|\widehat{\pmb \Theta }-\pmb \Theta |, \,\,\,\, MSEs=\frac{1}{K}\sum _{i=1}^{K}(\widehat{\pmb \Theta }-\pmb \Theta )^2, \,\,\,\, MREs=\frac{1}{K}\sum _{i=1}^{K}|\widehat{\pmb \Theta }-\pmb \Theta |/\pmb \Theta , \end{aligned}$$

where $\pmb \Theta$=($\alpha , \beta , \lambda ,\theta$). All calculations were performed by using the R software version 4.1.2.

Tables 4, 5 and 6 summarized the results of the simulation studies for the proposed model using the four estimation procedures. From the results, it can be concluded that as the sample size increases, all estimation methods of the proposed distribution approach to their initial guess of values. Furthermore, in all cases, the values of MSEs, and MREs tend to decrease. This ensures the consistency and asymptotically impartiality of all estimators. Additionally, by taking the MSE as an optimally criteria, we deduce that MLEs outperform alternative methods of estimate for the MPGWD.

**Table 4:** The ABs, MSEs and associated MREs of the ($\alpha , \beta , \lambda , \theta$)=(0.5, 0.4, 0.8, 0.9) considering different sample sizes.

*n*	Method	${\hat{\alpha }}$			${\hat{\beta }}$			${\hat{\lambda }}$			${\hat{\theta }}$		
		AB	MSE	MRE	AB	MSE	MRE	AB	MSE	MRE	AB	MSE	MRE
100	MLE	0.1508	0.0504	0.3016	0.3704	0.1997	0.9260	0.9702	2.6293	1.2128	0.1063	0.0492	0.1181
	LSE	0.0311	0.0963	0.0623	0.1275	0.2652	0.3187	0.9316	3.3506	1.1645	0.2485	0.8041	0.2761
	MPS	0.1352	0.0634	0.2704	0.8778	0.2184	0.8778	0.6210	2.9341	0.6210	0.6040	0.4631	0.6040
	CVE	0.0704	0.0868	0.1409	0.0650	0.2387	0.1625	0.4673	3.0700	0.5841	0.4040	0.7894	0.4489
300	MLE	0.0630	0.0287	0.3133	0.1714	0.1714	0.3750	0.0209	0.0669	0.0261	0.1266	0.0263	0.1406
	LSE	0.0140	0.0892	0.0280	0.0398	0.2178	0.0996	0.1526	0.5521	0.1908	0.0318	0.0957	0.0353
	MPS	0.5934	0.0591	0.5934	0.7894	0.1967	0.7894	0.1482	0.3674	0.1482	0.1649	0.0531	0.1649
	CVE	0.0639	0.0625	0.1279	0.0469	0.2037	0.1172	0.2940	0.5053	0.3676	0.0405	0.0763	0.0450
500	MLE	0.0355	0.0049	0.0710	0.3998	0.1599	0.9995	0.0624	0.0395	0.0780	0.0022	0.0014	0.0024
	LSE	0.0393	0.0715	0.0787	0.0353	0.1887	0.0884	0.0933	0.4449	0.1167	0.0391	0.0726	0.0434
	MPS	0.4841	0.0352	0.4814	0.6547	0.1678	0.6547	0.1165	0.2182	0.1165	0.0982	0.0293	0.0982
	CVE	0.0387	0.0512	0.0775	0.0375	0.1718	0.0939	0.1353	0.3843	0.1691	0.0432	0.0621	0.0480
700	MLE	0.0043	0.0019	0.0086	0.3969	0.1579	0.9923	0.0004	0.0281	0.0005	0.0053	0.0012	0.0058
	LSE	0.0513	0.0618	0.1027	0.0131	0.1763	0.0327	0.1494	0.3719	0.1868	0.0485	0.0408	0.0539
	MPS	0.3674	0.0162	0.3674	0.5867	0.1632	0.5867	0.0751	0.1791	0.0751	0.0822	0.0931	0.0822
	CVE	0.0647	0.0427	0.1295	0.0240	0.1657	0.0601	0.2248	0.1394	0.2810	0.0099	0.0345	0.0110
1000	MLE	0.0075	0.0008	0.0150	0.0193	0.1434	0.0483	0.1596	0.0141	0.1995	0.0040	0.0008	0.0044
	LSE	0.1035	0.0532	0.1035	0.0988	0.1687	0.0988	0.4756	0.3556	0.1872	0.0562	0.0349	0.0925
	MPS	0.2861	0.0110	0.2861	0.4298	0.1508	0.4298	0.0492	0.1271	0.0492	0.0718	0.0159	0.0478
	CVE	0.0474	0.0429	0.0948	0.0106	0.1530	0.0265	0.1498	0.3043	0.0875	0.0562	0.0341	0.0624

**Table 5:** The ABs, MSEs and associated MREs of the ($\alpha , \beta , \lambda , \theta$)=(1, 1, 1, 1) considering different sample sizes.

*n*	Method	${\hat{\alpha }}$			${\hat{\beta }}$			${\hat{\lambda }}$			${\hat{\theta }}$		
		AB	MSE	MRE	AB	MSE	MRE	AB	MSE	MRE	AB	MSE	MRE
100	MLE	0.4017	1.6641	0.4017	0.0998	0.0099	0.0998	0.0901	0.1019	0.0901	0.3142	1.2024	0.3142
	LSE	0.4397	3.6129	0.4397	0.2786	0.6793	0.2786	0.8019	7.4673	0.8019	0.1464	1.8019	0.1464
	MPS	0.6402	1.7358	0.6402	0.8778	0.4465	0.8778	0.6210	1.1844	0.6210	0.6040	1.2716	0.6040
	CVE	0.2054	2.0067	0.2054	0.3372	0.5912	0.3372	0.8548	6.8765	0.8548	0.1178	1.3331	0.1178
300	MLE	0.1871	0.6220	0.1871	0.0983	0.0097	0.0983	0.0016	0.0954	0.0016	0.2182	0.5500	0.2182
	LSE	0.1811	1.3889	0.1811	0.0998	0.5182	0.0998	0.7562	4.1285	0.7562	0.2965	1.1058	0.2965
	MPS	0.5934	0.7467	0.5934	0.7894	0.1988	0.7894	0.1482	0.1533	0.1482	0.1649	0.7462	0.1649
	CVE	0.0785	0.8707	0.0785	0.1539	0.2349	0.1539	0.5050	2.1604	0.5050	0.1081	0.8214	0.1081
500	MLE	0.0036	0.4411	0.0036	0.0980	0.0095	0.0980	0.0010	0.0891	0.0010	0.1394	0.1689	0.1394
	LSE	0.0533	0.7601	0.0533	0.0994	0.3293	0.0998	0.6432	2.2361	0.6432	0.2006	0.5527	0.2006
	MPS	0.4841	0.5247	0.4814	0.6547	0.1047	0.6547	0.1165	0.1672	0.1165	0.0982	0.3459	0.0982
	CVE	0.0437	0.7233	0.0437	0.1314	0.1442	0.1314	0.1604	1.8454	0.1604	0.0426	0.3504	0.0426
700	MLE	0.0032	0.2869	0.0076	0.0793	0.0076	0.0793	0.0007	0.0712	0.0007	0.0614	0.0566	0.0614
	LSE	0.1084	0.4487	0.1084	0.0991	0.2941	0.0991	0.5873	1.2705	0.5873	0.1098	0.1535	0.1098
	MPS	0.3674	0.3152	0.3674	0.5867	0.0854	0.5867	0.0751	0.1791	0.0751	0.0822	0.0931	0.0822
	CVE	0.0352	0.3990	0.0352	0.0982	0.1059	0.0982	0.0890	1.2390	0.0890	0.0408	0.1335	0.0408
1000	MLE	0.0023	0.2258	0.0023	0.0727	0.0071	0.0727	0.0002	0.0645	0.0002	0.0429	0.0478	0.0429
	LSE	0.1035	0.3632	0.1035	0.0988	0.2487	0.0988	0.4756	0.9256	0.4756	0.0925	0.1249	0.0925
	MPS	0.2861	0.2849	0.2861	0.4298	0.0.538	0.4298	0.0492	0.1821	0.0492	0.0718	0.1359	0.0478
	CVE	0.0050	0.3410	0.0050	0.0509	0.0981	0.0509	0.0875	0.0902	0.0875	0.0650	0.0664	0.0650

**Table 6:** The ABs, MSEs and associated MREs of the ($\alpha , \beta , \lambda , \theta$)=(0.75, 0.6, 0.7, 0.4) considering different sample sizes.

*n*	Method	${\hat{\alpha }}$			${\hat{\beta }}$			${\hat{\lambda }}$			${\hat{\theta }}$		
		AB	MSE	MRE	AB	MSE	MRE	AB	MSE	MRE	AB	MSE	MRE
100	MLE	0.1945	0.0874	0.4712	0.4621	0.2607	1.2442	1.3112	2.9281	1.3561	0.1494	0.0791	0.1711
	LSE	0.0771	0.1323	0.1431	0.1846	0.3202	0.3709	1.2517	4.2842	1.6532	0.3361	1.0373	0.3206
	MPS	0.1722	0.1009	0.3081	0.4649	0.2861	0.8293	0.9243	3.0373	0.8943	0.1892	0.1161	0.2231
	CVE	0.2036	0.1276	0.3482	0.4812	0.3018	0.8643	0.9726	3.0859	0.9248	0.2134	0.1515	0.2559
300	MLE	0.1734	0.0719	0.4119	0.4226	0.2312	0.9473	1.1257	1.5816	0.9647	0.1364	0.0526	0.1519
	LSE	0.0527	0.1137	0.1249	0.1573	0.2816	0.3485	0.9789	2.6248	1.3681	0.3025	0.7211	0.2529
	MPS	0.1473	0.0891	0.2714	0.4437	0.2550	0.7934	0.8946	1.6274	0.8167	0.1593	0.0813	0.1882
	CVE	0.1687	0.1045	0.3022	0.4367	0.2705	0.8152	0.9254	1.6516	0.8726	0.1859	0.1149	0.2161
500	MLE	0.1262	0.0557	0.3892	0.4019	0.2017	0.8961	1.0943	1.3385	0.8437	0.1049	0.0452	0.1227
	LSE	0.0513	0.0994	0.1018	0.1287	0.2664	0.3128	0.9561	2.2486	1.2673	0.2742	0.3716	0.2038
	MPS	0.1223	0.0694	0.2475	0.4170	0.2217	0.7528	0.8416	1.3612	0.7762	0.1236	0.0649	0.1568
	CVE	0.1337	0.0872	0.2719	0.3984	0.2470	0.7793	0.8648	1.3976	0.7842	0.1458	0.0937	0.1789
700	MLE	0.1384	0.0451	0.4012	0.4175	0.1634	0.9157	1.1105	0.8216	0.8624	0.1135	0.0338	0.1352
	LSE	0.0614	0.0819	0.1082	0.1367	0.2173	0.3254	0.9587	2.0917	1.2746	0.2783	0.2291	0.2074
	MPS	0.1362	0.0667	0.2749	0.4197	0.1782	0.7568	0.8469	0.8602	0.7801	0.1281	0.0416	0.1590
	CVE	0.1406	0.0773	0.2849	0.4027	0.1911	0.7853	0.8681	0.8894	0.8871	0.1462	0.0621	0.1816
1000	MLE	0.1120	0.0216	0.3617	0.3647	0.1482	0.8234	0.9328	0.6437	0.7365	0.0754	0.0094	0.0816
	LSE	0.0367	0.0731	0.0719	0.3652	0.1743	0.7634	0.8794	1.4672	0.9461	0.9321	0.1365	0.1682
	MPS	0.1024	0.0437	0.0863	0.3557	0.1627	0.2482	0.7892	0.6723	0.6938	0.0985	0.0169	0.1246
	CVE	0.1057	0.0620	0.2264	0.3576	0.1726	0.6894	0.8133	0.6982	0.7167	0.1293	0.0359	0.1205

## Real data analysis

Through performing goodness-of-fit tests, we utilize two data sets to contrast the MPGW model with PGW distribution and the other four alternative existing models to see the effectiveness of the new model. The compared distributions:

1. Additive modified Weibull (AMW) distribution^4^ with pdf defined as follows
  $$\begin{aligned} g\left( x;\lambda ,\theta ,\alpha ,\beta \right) =\left( \alpha \theta x^{\theta -1} +\lambda \beta x^{\lambda -1} \right) \exp \left( -\alpha x^{\theta } -\beta x^{\lambda } \right) ;\, \, x\ge 0,\, \, \alpha ,\beta \ge 0,\theta \mathrm {>}0,0\mathrm {<}\lambda \mathrm {<}1. \end{aligned}$$
2. Modified extension Weibull (MEW) distribution^21^ with pdf defined as follows
  $$\begin{aligned} g\left( x;\lambda ,\theta ,\beta \right) =\lambda \beta \left( \theta x\right) ^{\beta -1} \exp \left[ \left( \theta x\right) ^{\beta } +\frac{\lambda }{\theta } \left( 1-e^{\left( \theta x\right) ^{\beta } } \right) \right] ,\, \, \, x\mathrm {>}0,\, \lambda ,\theta ,\beta \mathrm {>}0. \end{aligned}$$
3. Extended Weibull (EW) distribution^22^ with pdf defined as follows
  $$\begin{aligned} g\left( x;\lambda ,\alpha ,\beta \right) =\alpha \left( \lambda +\beta x\right) x^{\beta -2} \exp \left( -\lambda /x-\alpha x^{\beta } e^{-\lambda /x} \right) ,\, \, \, x\mathrm {>}0,\, \, \alpha ,\lambda ,\beta \mathrm {>}0. \end{aligned}$$
4. Flexible Weibull (FW) distribution^5^ with pdf defined as follows
  $$\begin{aligned} g\left( x;\alpha ,\beta \right) =\left( \alpha +\beta /x^{2} \right) \exp \left( \alpha x-\beta /x-e^{\alpha x-\beta /x} \right) ;x,\alpha ,\beta \mathrm {>}0. \end{aligned}$$
5. Kumaraswamy Weibull (KW) distribution^23^ with pdf defined as follows
  $$\begin{aligned} g\left( x;\lambda ,\theta ,\alpha ,\beta \right) =\alpha \beta \theta \lambda e^{-(\lambda x)^\theta } (\lambda x)^{\theta -1} \left( 1-e^{-(\lambda x)^\theta }\right) ^{\alpha -1} \left( 1-\left( 1-e^{-(\lambda x)^\theta }\right) ^\alpha \right) ^{\beta -1};x,\alpha ,\beta ,\theta ,\lambda >0. \end{aligned}$$
6. Beta Weibull (BW) distribution^24^ with pdf defined as follows
  $$\begin{aligned} g\left( x;\lambda ,\theta ,\alpha ,\beta \right) =\frac{\theta (x/\alpha )^{\theta -1}}{\alpha B(\alpha ,\beta )}(1-e^{-(x/\alpha )^{\theta }})^{\alpha -1} e^{-\beta (x/\alpha )^{\theta }};x,\alpha ,\beta ,\theta ,\alpha >0. \end{aligned}$$

The first data set represents the recorded remission times given in months from bladder cancer patients, reported by Lee and Wang^25^. The ordered array of the data is

**Table:** 

0.08	1.35	2.46	3.25	3.88	4.98	5.62	7.26	8.26	10.34	12.63	17.12	25.82
0.2	1.4	2.54	3.31	4.18	5.06	5.71	7.28	8.37	10.66	13.11	17.14	26.31
0.4	1.46	2.62	3.36	4.23	5.09	5.85	7.32	8.53	10.75	13.29	17.36	32.15
0.5	1.76	2.64	3.36	4.26	5.17	6.25	7.39	8.65	11.25	13.8	18.1	34.26
0.51	2.02	2.69	3.48	4.33	5.32	6.54	7.59	8.66	11.64	14.24	19.13	36.66
0.81	2.02	2.69	3.52	4.34	5.32	6.76	7.62	9.02	11.79	14.76	20.28	43.01
0.9	2.07	2.75	3.57	4.4	5.34	6.93	7.63	9.22	11.98	14.77	21.73	46.12
1.05	2.09	2.83	3.64	4.5	5.41	6.94	7.66	9.47	12.02	14.83	22.69	79.05
1.19	2.23	2.87	3.7	4.51	5.41	6.97	7.87	9.74	12.03	15.96	23.63	
1.26	2.26	3.02	3.82	4.87	5.49	7.09	7.93	10.06	12.07	16.62	25.74	

The second data set considered the values of the survival times given in days of guinea pigs infected with virulent tubercle bacilli, summarized by Bjerkedal^14^. The ordered array of the data is

**Table:** 

0.1	0.74	1	1.08	1.16	1.3	1.53	1.71	1.97	2.3	2.54	3.47
0.33	0.77	1.02	1.08	1.2	1.34	1.59	1.72	2.02	2.31	2.54	3.61
0.44	0.92	1.05	1.09	1.21	1.36	1.6	1.76	2.13	2.4	2.78	4.02
0.56	0.93	1.07	1.12	1.22	1.39	1.63	1.83	2.15	2.45	2.93	4.32
0.59	0.96	1.07	1.13	1.22	1.44	1.63	1.95	2.16	2.51	3.27	4.58
0.72	1	1.08	1.15	1.24	1.46	1.68	1.96	2.22	2.53	3.42	5.55

Table 7 recorded different statistic measures for the two proposed data sets.

**Table 7:** Basic statistics for the two data.

Considered data set	Min.	Qu${}_{1}$	Qu${}_{2}$	Mean.	Qu${}_{.}$	Std.	$\beta _1$	$\beta _2$	Max.
First data set	0.08	3.35	6.40	9.37	11.84	10.51	3.25	15.20	79.05
Second data set	0.10	1.08	1.40	1.77	2.24	1.03	1.31	1.85	5.55

To assess the validity of the proposed model, we conducted several statistical tests and computed various criterion measures. Firstly, we computed the log-likelihood function (-L), then, we employed criterion measures such as the Akaike Information Criterion ($\mathcal {A}_1$) and the Bayesian Information Criterion ($\mathcal {B}_1$) to evaluate the performance of the model further. The model that yields the minimum values of these criteria is considered to be the most appropriate for the given data set. To complement the criterion measures, we also employed various test statistics, including the Cramér-von Mises (Cr), Anderson–Darling (An), and Kolmogorov–Smirnov (KS) tests. These tests assess the model’s overall fit by comparing the observed data with the model’s predicted values. The associated p-values obtained from these tests measure the statistical significance of the differences between the observed and predicted values. By considering these criterion measures and test statistics, we can comprehensively evaluate the validity of the proposed model. The model that exhibits the best fit, as indicated by the minimum values of the criterion measures and non-significant p-values from the test statistics, can be considered the most suitable for the given data set.

**Table 8:** The MLEs and corresponding L, $\mathcal {A}_1$ and $\mathcal {B}_1$ values for different fitting models using first data.

Distributions	Parameters(SE)				L	$\mathcal {A}_1$	$\mathcal {B}_1$
	${\widehat{\lambda }}$	${\widehat{\theta }}$	${\widehat{\alpha }}$	${\widehat{\beta }}$			
MPGW	0.0233	3.5251	0.1153	0.0520	$-409.3373$	826.6746	838.0827
	(0.0575668)	(2.70518)	(0.123153)	(0.0236309)			
PGW	0.1416	1.5568	0.4222	–	$-410.3021$	826.6042	835.1603
	(0.0394446)	(0.240679)	(0.1092)				
AMW	0.9729	1.0478	0.0939	1.10E-8	$-414.0869$	836.1738	847.5819
	(0.10497)	(0.067576)	(0.019081)	(0.0000498)			
MEW	4.04E3	4.53E7	–	0.1119	$-419.7096$	845.4192	853.9753
	(3338.68)	(6.3942E7)		(0.004758)			
EW	0.1271	–	0.1026	1.0197	$-413.5593$	833.1187	841.6748
	(0.153653)		(0.006095)	(0.002585)			
FW	–	–	0.0325	2.1548	$-460.2659$	924.5318	930.2358
			(0.002555)	(0.248939)			
KW	0.2159	0.4589	4.1178	2.9414	$-410.5691$	829.1382	840.5463
	(0.249025)	(0.515299)	(5.83644)	(8.14823)			
BW	3.1098	0.6661	2.7348	0.9083	$-410.6786$	829.3571	840.7653
	(0.51804)	(0.242903)	(6.46409)	(0.825413)

**Table 9:** The MLEs and corresponding L, $\mathcal {A}_1$ and $\mathcal {B}_1$ values for different fitting models using second data.

Distributions	Parameters(SE)				L	$\mathcal {A}_1$	$\mathcal {B}_1$
	${\widehat{\lambda }}$	${\widehat{\theta }}$	${\widehat{\alpha }}$	${\widehat{\beta }}$			
MPGW	1.77E11	296.2071	0.0029	0.1266	$-89.23162$	186.4632	195.5699
	(46316.7)	(46.116)	(0.0032747)	(0.0452335)			
PGW	0.8170	2.8420	0.3880	–	$-93.53814$	193.0763	199.9063
	(0.412897)	(0.621165)	(0.135424)				
AMW	0.3710	1.8254	0.2832	1.10E-8	$-95.78981$	199.5796	208.6863
	(0.270093)	(0.158711)	(0.0540902)	(0.0000231)			
MEW	154.8199	0.0005	–	1.8255	$-95.78984$	197.5797	204.4097
	(217.692)	(0.00098)		(0.469472)			
EW	0.1643	–	0.3373	1.7175	$-95.56381$	197.1276	203.9576
	(0.280029)		(0.117196)	(0.237983)			
FW	–	–	0.3824	1.4381	$-102.4335$	208.8670	213.4203
			(0.036711)	(0.178066)			
KW	0.7665	0.9913	3.1103	1.7319	$-94.0656$	196.1312	205.2379
	(0.702075)	(1.04436)	(3.86848)	(5.52747)			
BW	0.8673	1.2907	2.3572	0.5414	$-94.0368$	196.0735	205.1802
	(0.616053)	(0.454981)	(1.37574)	(0.871254)

**Table 10:** The goodness of fit test for various fitting distributions by applying the first data set.

Models	Cr	An	KS	p-Cr	p-An	p-KS
MPGW	0.0145	0.0971	0.0313	0.9997	0.9999	0.9996
GW	0.0311	0.2134	0.0390	0.9727	0.9863	0.9900
AMW	0.1537	0.9577	0.0700	0.3789	0.3801	0.5570
MEW	0.3285	1.9867	0.0958	0.1125	0.0935	0.1910
EW	0.1400	0.8754	0.0698	0.4219	0.4295	0.5617
FW	1.5915	8.1568	0.2084	9.94E-05	9.81E-05	2.97E-05
KW	0.0378	0.2544	0.0447	0.9445	0.9680	0.9603
BW	0.0403	0.2704	0.0450	0.9320	0.9586	0.9582

**Table 11:** The goodness of fit test for various fitting distributions by applying the second data set.

Models	Cr	An	KS	p-Cr	p-An	p-KS
MPGW	0.0301	0.1915	0.0531	0.9767	0.9926	0.9873
GW	0.0693	0.4361	0.0807	0.7575	0.8118	0.7370
AMW	0.1680	1.0072	0.1048	0.3397	0.3533	0.4079
MEW	0.1680	1.0071	0.1048	0.3397	0.3533	0.4082
EW	0.1484	0.9073	0.1058	0.3953	0.4094	0.3964
FW	0.2216	1.4288	0.1464	0.2295	0.1944	0.0912
KW	0.0858	0.5354	0.0890	0.6605	0.7104	0.6189
BW	0.0859	0.5346	0.0886	0.6599	0.7112	0.6241

**Figure 3:**
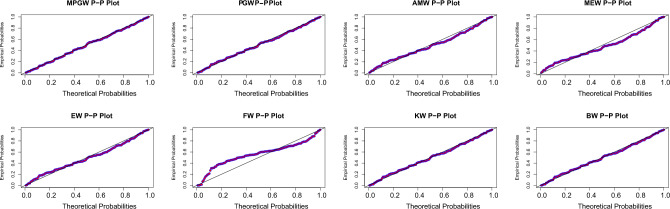
P-P plots of MPGW, GW, AMW, MEW, EW, FW, KW, and BW for the first data set.

**Figure 4:**
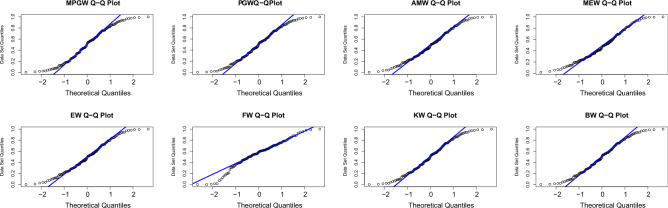
QQ plots of MPGW, GW, AMW, MEW, EW, FW, KW, and BW for the first data set.

**Figure 5:**
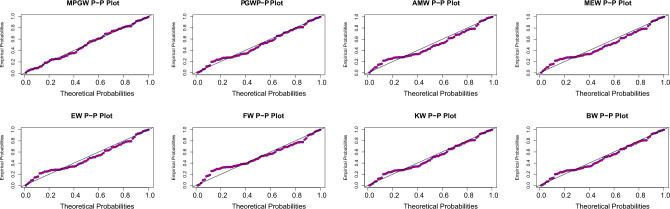
P-P plots of MPGW, GW, AMW, MEW, EW, FW, KW, and BW for the second data set.

**Figure 6:**
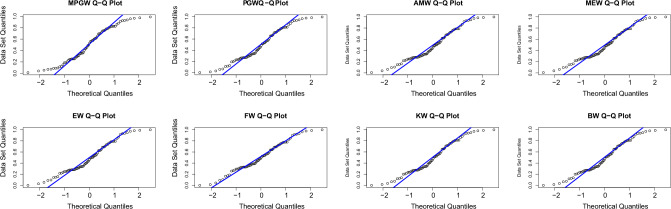
QQ plots of MPGW, GW, AMW, MEW, EW, FW, KW, and BW for the second data set.

Tables 8 and 9, contain the values of criterion measure statistics for the fitted models by applying the two considered data sets. Based on these measures and along with the *p*-values of the proposed test statistics for each distribution, the MPGW model is the best candidate distribution for modeling the two data sets. The plots of the probability–probability (P–P) and quartile–quartile (Q–Q) of the suggested distributions using the two proposed data are shown in Figs. 3, 4, 5 and 6. This figure confirms this conclusion.

**Figure 7:**
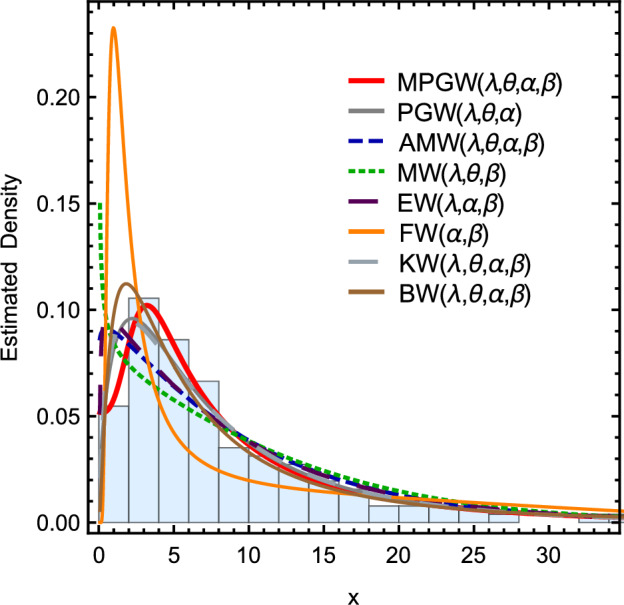
Curves of the pdfs for different fitting distributions using the first data set.

**Figure 8:**
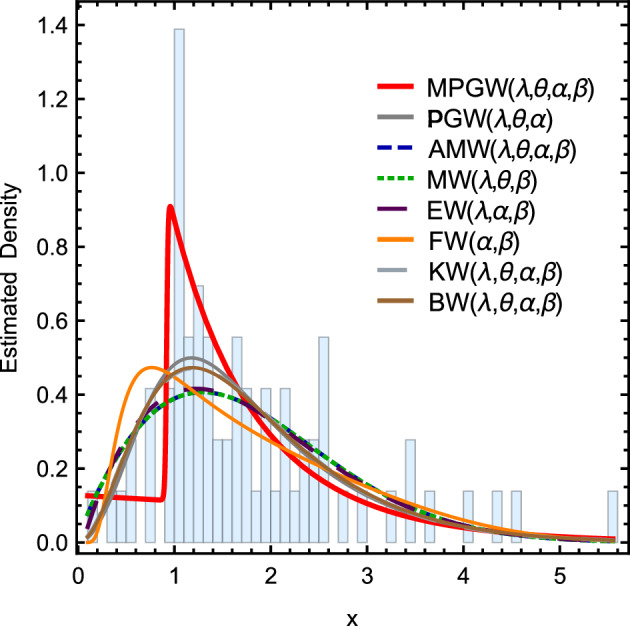
Curves of the pdfs for different fitting distributions using the second data set.

Figure 7 shows the curves of the pdfs for different fitting distributions using the first data set. Figure 8 shows the Curves of the pdfs for different fitting distributions using the second data set. Tables 10 and 11 contain The goodness of fit test for various fitting distributions by applying the first and second data sets, respectively.

## Conclusion

This research paper introduces a novel distribution that involves compounding two cumulative hazard rate functions. We have derived a specific sub-model from the proposed distribution and established various mathematical properties related to it. We have applied four different estimation techniques to estimate the unknown parameters of our suggested model. Additionally, we have conducted simulation experiments to evaluate the effectiveness of these proposed estimation methods. Furthermore, we have analyzed two real engineering data sets to assess how well the MPGW model fits the data when compared to other well-known models. Our findings indicate that the MPGW model demonstrates a good fit to the data sets, highlighting its potential utility in practical applications.

Looking ahead, there are several potential avenues for future research. Firstly, we can extend our work to study the bivariate case and explore different properties of the proposed distribution within that context. Additionally, we can investigate the application of different censored methods, such as progressive type I, II, and hybrid censored methods, for estimating the unknown parameters of the proposed model. Moreover, we may explore the estimation of model parameters using Bayesian approaches and consider various loss functions, such as square error, Linex, and general entropy, to further enhance our understanding of the proposed model. The current study can be extended using neutrosophic statistics as future research; see^26–28^.

## Data Availability

All references exist in the paper for data used in the paper; see Lee and Wang^25^ for the first real data set and Bjerkedal^14^ for the second one.
